# Supplementary material for: Parliamentary roll-call voting as a complex dynamical system: The case of Chile
Source: PLoS One. 2023 Apr 26;18(4):e0281837. doi: 10.1371/journal.pone.0281837 (PMC10132531; doi:10.1371/journal.pone.0281837)
Supplement: S1 Appendix — (DOCX) [file pone.0281837.s001.docx]

# S1 Appendix A. Example of a Recurrence Plot (RP) for the 2006-2010 legislative period.

A recurrence plot for the agreement ratio in the 2006-2010 term is shown (Fig. 1). This period is contrasted with a recurrence plot of the data for the same period but in random order. We can see that the regularities observed with the data in the original order are lost by randomizing the order of observations. The recurrence rate (density of recurrence points in a recurrence plot) and determinism (diagonal lines in the recurrence plot of a minimal length) are calculated from the recurrence plot, among other metrics.


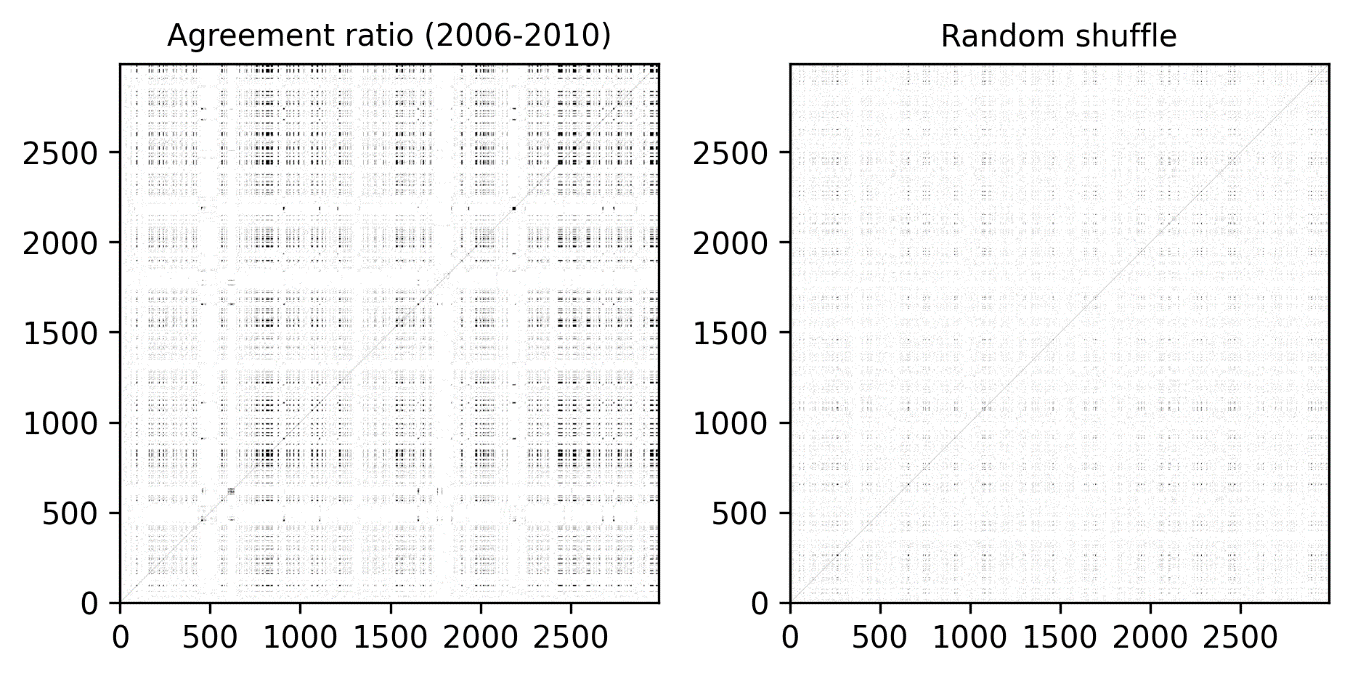


**Fig. 1** Recurrence plot for the agreement ratio of the 2006-2010 term. On the left is the plot obtained with the data in the original temporal order, and on the right is the plot with the data randomly sorted. These plots were produced with a delay of 1, 6 embedded dimensions, and a threshold of 0.17.
