# Supplementary material for: Parliamentary roll-call voting as a complex dynamical system: The case of Chile
Source: PLoS One. 2023 Apr 26;18(4):e0281837. doi: 10.1371/journal.pone.0281837 (PMC10132531; doi:10.1371/journal.pone.0281837)
Supplement: S2 Appendix — (DOCX) [file pone.0281837.s002.docx]

# S2 Appendix B. Composition of the Chamber of Deputies regarding what percentage is considered opposition and what percentage supports the government in each period.

The political composition of the Chamber of Deputies was analyzed for each period. We estimated what percentage is considered opposition and what percentage supports the government in each legislative period. This estimate was done by observing the political coalitions of each legislative period and whether that coalition had a similar political tendency to the government in that period.

Table 1 shows that the 2014-2018 period had the highest percentage of deputies who support the government (59.17%), while the 2010-2014 period had the lowest percentage (49.17%). These percentages should only be considered as an estimate since, given the multiparty system of the Chilean political structure, the discipline of political parties to their coalition is not always stable.

| **Table 1** Estimated percentage of deputies supportive of the government and percentage considered as opposition | | |
| --- | --- | --- |
| **Legislative Period** | **Government Supporters (%)** | **Opposition (%)** |
| 2002-2006 | 51.67% | 47.50% |
| 2006-2010 | 54.20% | 45.80% |
| 2010-2014 | 49.17% | 50.83% |
| 2014-2018 | 59.17% | 40.80% |
| 2018-2021 | 51.60% | 48.40% |
