# Supplementary material for: Parliamentary roll-call voting as a complex dynamical system: The case of Chile
Source: PLoS One. 2023 Apr 26;18(4):e0281837. doi: 10.1371/journal.pone.0281837 (PMC10132531; doi:10.1371/journal.pone.0281837)
Supplement: S3 Appendix — (DOCX) [file pone.0281837.s003.docx]

# S3 Appendix C. Detrended Fluctuation Analysis (DFA).

To study the self-affinity of the data, i.e., the presence of repeating patterns at different time scales, a Detrended Fluctuation Analysis (DFA) was performed. DFA provides an exponent like the Hurst exponent, whose value is informative of the time series autocorrelation. A value around 0.5 indicates the presence of uncorrelated or Gaussian white noise in the time series. A value greater than 0.5 indicates that the time series are positively correlated. A value of approximately 1 indicates a 1/f-noise distribution or pink noise (a signal with long-range dependence).

The results indicated that the agreement ratio (α = 0.73, R² = 0.94) and voting outcome (α = 0.68, R² = 0.93) were positively correlated in time, suggesting that the system has long-range memory. Next, the agreement ratio and voting outcome were divided by legislative periods. The results showed that this property remained relatively stable in each period (Fig. 1). In the agreement ratio (Fig. 1, Panel A), the variation of the exponent in each term was small (SD = 0.03), with some decay in the last period. The voting outcome (Fig. 1, Panel B) remained relatively stable between legislative periods (SD = 0.06); however, a decay in the exponent was seen during the 2010-2014 term. As expected, when randomly shuffling each time series, the exponent obtained equals 0.5, corresponding to white noise. In conclusion, these results indicate that despite variations in the data, they remain positively correlated over time.


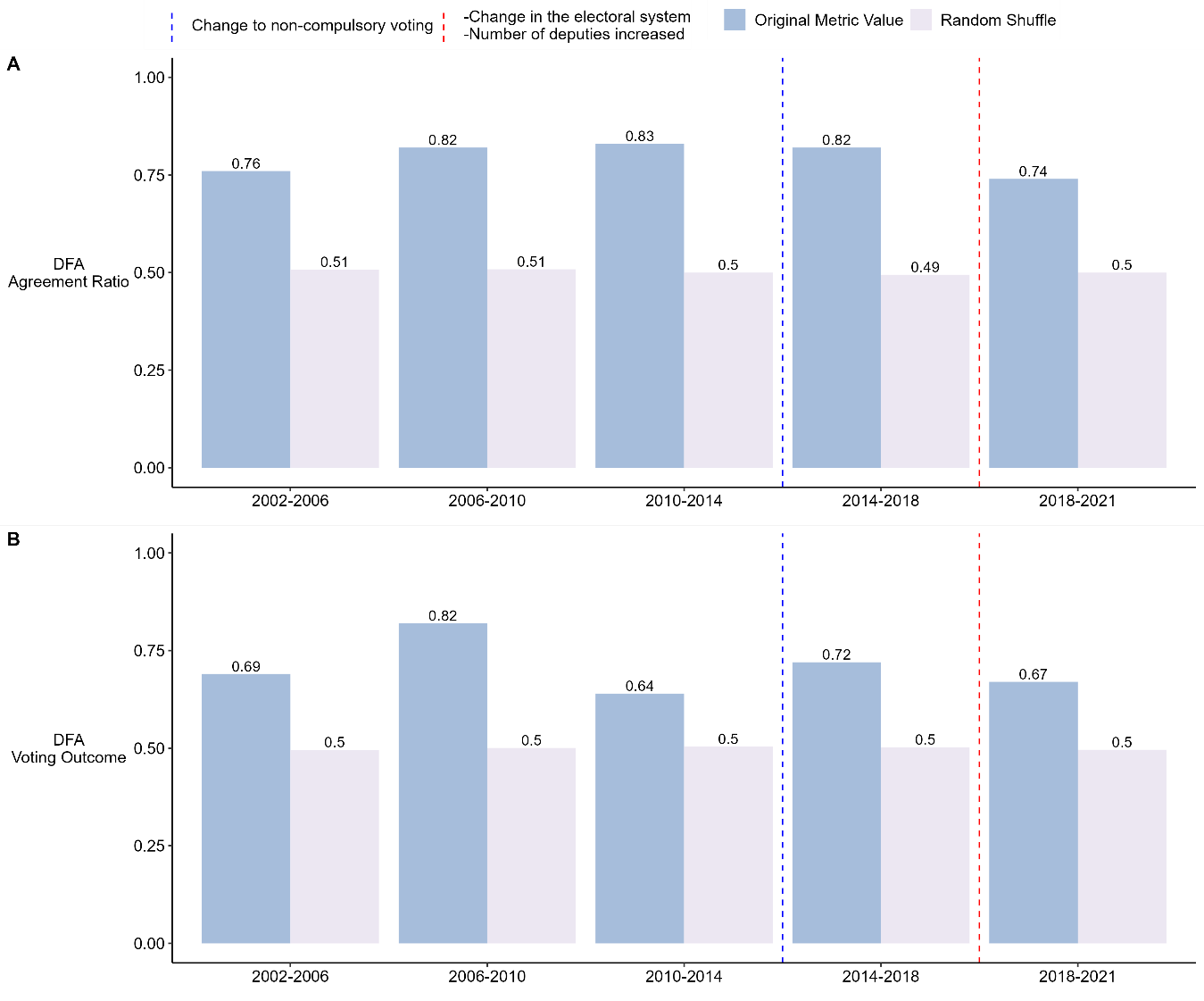


**Fig. 1** Detrended Fluctuation Analysis of the Agreement Ratio (Panel A) and Voting Outcome (Panel B) for each legislative period. The result obtained with the original data series is shown, along with the average result of randomly shuffling the data for each period 30 times and performing the analyses on each shuffle. The blue dashed vertical line indicates when the first parliamentary elections with non-compulsory voting occurred. The red dashed vertical line indicates the first parliamentary elections with a propositional election system and the increase in the number of deputies.
