# Supplementary material for: Parliamentary roll-call voting as a complex dynamical system: The case of Chile
Source: PLoS One. 2023 Apr 26;18(4):e0281837. doi: 10.1371/journal.pone.0281837 (PMC10132531; doi:10.1371/journal.pone.0281837)
Supplement: S4 Appendix — (DOCX) [file pone.0281837.s004.docx]

# S4 Appendix D. Analyses to study if our data exhibits memory, regime shifts, and sensitive dependence on initial conditions.

We search for complexity markers using Olthof et al. analyses [1] to determine whether our data exhibit the following characteristics of complex systems: Memory (Dependency on past values, long-range temporal correlations, non-stationary temporal correlations), regime shifts (non-stationarity), and sensitive dependence on initial conditions (limited predictive horizon).

**Memory - Dependency on past values:** To identify whether the system has memory, i.e., depends on past values and is not random, we tested for randomness with the Bartels Rank Test (Bartels, 1982) using the "randtests" R package [2], on the agreement ratio and the voting outcome.

**Memory - Long-range temporal correlations:** To identify whether the system has long-term memory, we used the partial autocorrelation function (PACF), which shows the correlation of a time series with a lagged version of itself for different lag values. PACF corrects for autocorrelation of higher lags with lower lags. We used the pacf() function from the R-package "stats". The significance threshold was based on a two-tailed Z test with the time series length as the number of observations. Significant partial autocorrelations at high lags indicate long-range temporal correlations.

**Memory - Non-stationary temporal correlations:** Non-stationary temporal correlations were tested with a time-varying autoregressive (TV-AR) model at lag 1 [3] using the R-package MGCV. The model tests whether the smoothing time-varying function is different from zero. The TV-AR model's effective degrees of freedom (EDF) indicate the number of parameters needed to represent the smoothing function. An EDF of 2 indicates that the autocorrelation is not changing or is changing linearly. An EDF higher than 2 indicates that the autocorrelation is non-stationary.

**Regime shifts:** We tested whether the series has distributional changes, e.g., changes in the mean and variance. Statistically, this leads to non-stationarity. We used the KPSS test from the R-package "tseries" [4], which has the null hypothesis that the time series is stationary around a level. The alternative hypothesis is that there is a presence of a unit root: an unpredictable systematic pattern, indicating non-stationarity. Also, we estimated the number of regime shifts with a change-point analysis, using the e.divisive algorithm from the R-package "epc" [5], which identifies significant changes in the data distribution over time. The algorithm compares all data segments and tests for differences in significance using a permutation test. Multiple change points suggest that a time series is non-stationary.

**Sensitive dependence on initial conditions - Forecast skill:** Since complex systems tend to produce predictable trajectories only in the short term, predictability can be used to distinguish between different systems, i.e., random systems have no predictability. Simple deterministic systems are predictable in both the short and long term. The predictability of complex systems tends to decay over time. This characteristic is a result of nonlinearity. To test this, we computed the forecast skill (the correlation coefficient between predicted and actual values) based on the procedure proposed by Sugihara and May [6] using the R-package rEDM [7]. The data were divided into two parts of 2048 points; the first was for the data library and the other for prediction. The "EmbedDimension" function estimated that an embedding dimension of 2 was ideal.

We found that the agreement ratio and voting outcome exhibited characteristics of complex dynamical systems. That is: Memory (Dependency on past values, long-range temporal correlations, non-stationary temporal correlations; Table 1), regime shifts (non-stationarity; Table 1), and sensitive dependence on initial conditions (limited predictive horizon; Fig. 1).

| Table 1 Analysis of markers of complex systems for the agreement ratio and voting outcome. | | | | | | |
| --- | --- | --- | --- | --- | --- | --- |
|  | **Bartels rank test (H0 = random, H1 = non-random)** | **Significant Partial Autocorrelation** | | **TV-AR** | **KPSS test (H0 = level stationary, H1 = unit root)** | **change point detection (e.divisive)** |
| Data | p-value | N | Max Lag | EDF | p-value | Number of significant change points |
| Agreement Ratio | < .001* | 188 | 13347 | 9.77* | > .01* | 149 |
| Voting Outcome | < .001* | 212 | 9942 | 9.81* | > .01* | 153 |

Regarding the predictive horizon, the results indicated that the forecast skill decreased over time, as expected for series with characteristics of complex dynamical systems. Fig. 1 shows the forecast skill plotted against time for the agreement ratio (Fig. 1, Panel A) and voting outcome (Fig. 1, Panel B). The same plot for a sine wave signal (Fig. 1, Panel C) and a random uniform noise signal (Fig. 1, Panel D) are shown for contrast.


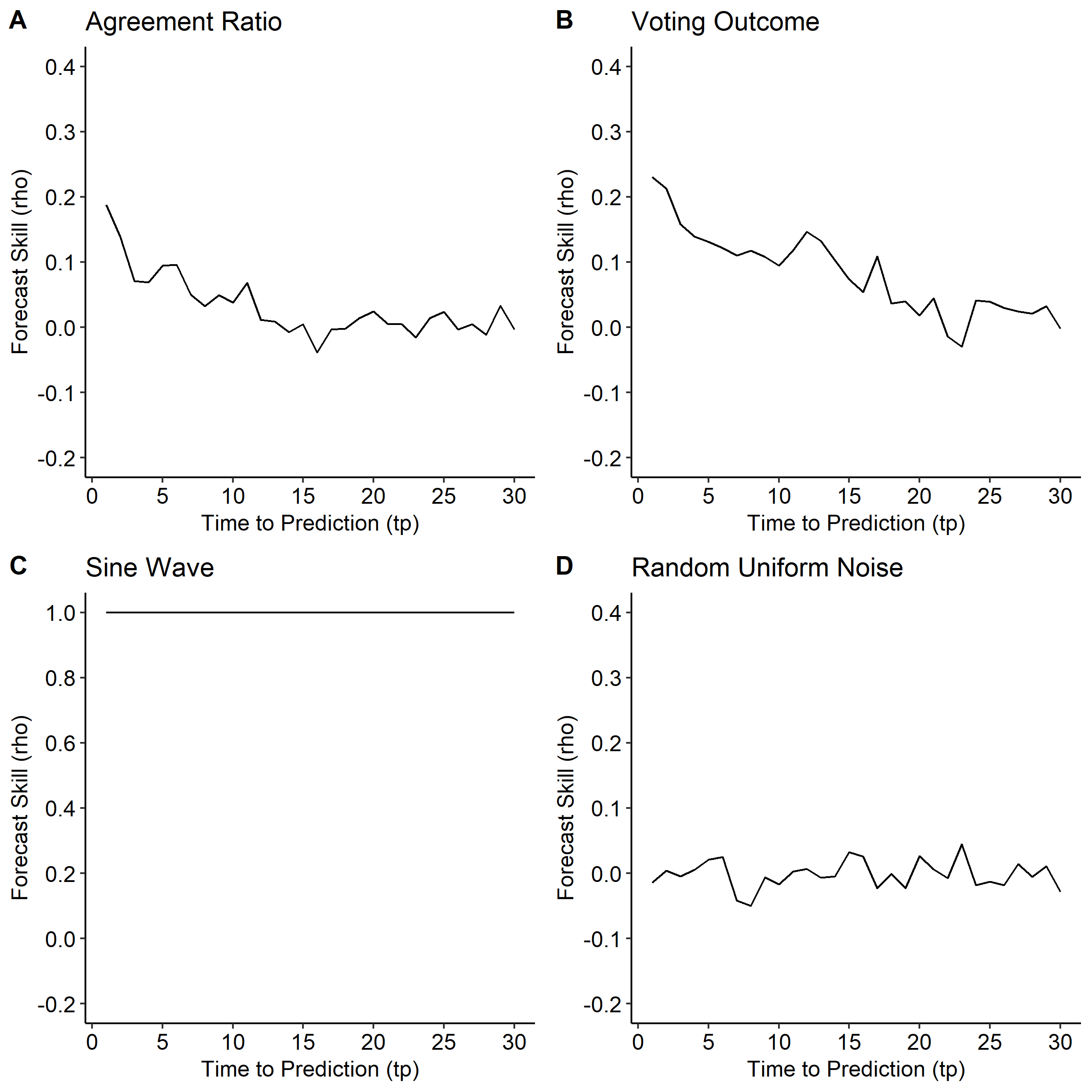


**Fig. 1** Forecast skill (correlation coefficient between predicted and actual values) over time plotted for the agreement ratio (Panel A), voting outcome (Panel B), a sine wave (Panel C), and random uniform noise (Panel D).

# References

[1] Olthof M, Hasselman F, Lichtwarck-Aschoff A. Complexity in psychological self-ratings: implications for research and practice. BMC Med 2020;18:1–16. https://doi.org/10.1186/s12916-020-01727-2.

[2] Mateus A, Caeiro F. An R implementation of several randomness tests, Athens, Greece: 2014, p. 531–4. https://doi.org/10.1063/1.4897792.

[3] Wood SN. Generalized additive models: an introduction with R. Second edition. Boca Raton: CRC Press/Taylor & Francis Group; 2017.

[4] Trapletti A, Hornik K. tseries: time series analysis and computational finance 2019.

[5] James NA, Matteson DS. ecp: An R package for nonparametric multiple change point analysis of multivariate data. J Stat Softw 2015;62. https://doi.org/10.18637/jss.v062.i07.

[6] Sugihara G, May RM. Nonlinear forecasting as a way of distinguishing chaos from measurement error in time series. Nature 1990;344:734–41. https://doi.org/10.1038/344734a0.

[7] Sugihara G, Park J, Ye H, Saberski E, Smith C. Empirical Dynamic Modeling 2020.
